# Trousseau on the Indications of Anemia

**Published:** 1845-12

**Authors:** 


*M. Trousseau on the indications of Anæmia.*—In the Journal de Médecine we find an interesting article by M. Trousseau, on anæmia, and the different indications of treatment which it presents. With many physicians, says M. Trosseau, anæmia is the same affection, whatever may be the cause which produces it, as in all cases it is constituted by an impoverished state of the blood, by predominance of the serosity, and diminution of the globules, the fibrin remaining in normal proportions. (This latter assertion is not correct, as the proportion of fibrin in anæmia is generally less than in the healthy state. It is only in chlorosis that it usually remains normal. See Simon's "Animal Chemistry," p. 306, et seq.)

The anæmia, or impoverished state of the blood being, thus, the principal symptom, the indication appears, at the first glance, to be very simple—viz: to re-constitute the blood by tonics, and preparations of iron. In reality, however, the disordered state of the blood, as appreciated by analysis, is only an element of the disease. There are other conditions of a more recondite nature, which must be taken into consideration. For instance, the state of the blood appears to be the same in a person who has suffered from hæmorrhage for a few days, as in one who has thus suffered for a great length of time. Yet, in the first case, the blood soon recovers its normal constitution, without the assistance of any therapeutic agent, whereas, in the second, the anæmia appears to have assumed a chronic form, and is often very difficult to cure. If the anæmia were all the disease, its duration would not thus modify its nature. In the latter instance, it is evident that the organs, long deprived of their accustomed stimulus, become deeply modified. Thus, anæmia from loss of blood may become a state of disease comparable to chlorosis. In chlorosis, the anæmia is evidently only a part of the disease, for although it gives way with a deceptive rapidity under the influence of analeptics and iron, it often returns without any cause, or under the influence of the most insignificent circumstance, and is frequently preceded and accompanied by various abnormal nervous symptoms, which are not observed in other anæmic diseases. It may also persist for years, notwithstanding the best directed treatment.

In anæmia from hæmorrhage, and in that of chlorosis, the treatment is the same, but anæmia is often the result of an organic lesion, and then the restorative treatment may be useless, or even do harm instead of good.

In the anæmia of cancerous disease iron is of no use, unless the cancerous diathesis is modified by loss of blood. In some forms of cancer—in uterine cancer, for instance—the anæmia of hæmorrhage may be superadded to that of the organic affection, in which case the administration of iron will often produce a wonderful change for the better.

In the purely cancerous anæmia, iron, if it does no good, does no harm. But this is not the case, says M. Trousseau, in the anæmia of albuminuria and of tubercles. Ferruginous preparations are often given in the first period of albuminaria, under the impression that the patient is laboring under chlorosis. But instead of bringing the blood back to its natural composition, they increase the predisposition to phlegmasiæ, so common in that disease, and render the fever, which sometimes accompanies albuminuria, more violent. M. Rayer, it is true, advises the use of ferruginous formulæ, but he must have been led to employ them more from a consideration of the composition of the blood than from clinical experience. With reference to tubercles, they produce anæmia, but a different kind of anæmia to that of chlorosis. There appears, indeed, to be a kind of antagonism between phthisis and chlorosis, so much so, that tubercles seldom make their appearance in chlorotic patients. Therapeutics still further demonstrate this difference. Iron modifies favorably chlorotic anæmia whilst it exercises a very unfavorable influence on tubercular anæmia. Thus, if iron is administered, as it often is, to young anæmic persons, born of scrofulous parents, and approaching the age of puberty, it will often determine the explosion of the tubercular cachexia, owing to its restoring to the blood its exciting properties, and thus developing the pulmonary phlegmasiæ which so often precede the manifestation and softening of tubercles. The chlorotic poverty of the blood of those who are predisposed to tubercles, is a guarantee of immunity from phthisis in this sense, that it renders these pulmonary phlegmasiæ less frequent. The antagonism which exists between phthisis and intermittent fever, and which has been pointed out of late years, is worthy of notice, in connection with that of chlorosis and tubercles. This antagonism is only observed when the miasmatic infection is carried to such an extent as to give rise to cachexia, or, in other words, to miasmatic anæmia. In this latter form of anæmia, iron is of no use, whereas quinine, or bark itself, properly administered, restores the blood to its normal color, and re-establishes the general health.

The same remarks apply to syphilitical anæmia, in which iron is powerless, whereas mercury generally succeeds in restoring the blood to a normal state, and in renovating the economy.—*Ibid.*

